# In Response

**DOI:** 10.4269/ajtmh.15-0524b

**Published:** 2015-12-09

**Authors:** Keun Hwa Lee, Jolyon M. Medlock, Yeojun Yun, Nam-Hyuk Cho

**Affiliations:** Department of Microbiology and Immunology Jeju National University School of Medicine Jeju, South Korea E-mail: yomust7@jejunu.ac.kr; Medical Entomology and Zoonoses Ecology Microbial Risk Assessment, Emergency Response Department Public Health England Wiltshire, United Kingdom E-mail: jolyon.medlock@phe.gov.uk; Ewha Medical Research Institute Ewha Womans University Seoul, South Korea E-mail: yyun@ewha.ac.kr; Department of Microbiology and Immunology Department of Biomedical Science Seoul National University College of Medicine Seoul, South Korea E-mail: chonh@snu.ac.kr

Dear Sir:

As with other emerging vector-borne diseases, there are many theories about the eco-epidemiology of severe fever with thrombocytopenia syndrome virus (SFTSV).

In our article, we highlighted the need for further work on the eco-epidemiology of SFTSV in Korea, and use Crimean–Congo hemorrhagic fever virus as an example of the role that migratory birds may play.

There is evidence that several factors might be involved in transmission, and the movement of infected ticks by birds cannot be overlooked.

Other infectious diseases such as Lyme borreliosis and tick-borne encephalitis move around Europe associated with birds infested with infected ticks. Similarly, the spread of West Nile virus in North America and highly pathogenic avian influenza virus H5N1 in east Asia was facilitated partly by bird movements.

We agree, however, that many factors may interact to explain the current distribution of SFTSV. One route of dissemination may not explain all incidences of disease.

We welcome the comments made in this letter^ 1^ as they highlight the need for further research.
